# Mood and Stress Evaluation of Adult Patients With Moyamoya Disease in Korea: Ecological Momentary Assessment Method Using a Mobile Phone App

**DOI:** 10.2196/17034

**Published:** 2020-05-25

**Authors:** Yong Sook Yang, Gi Wook Ryu, Chang Gi Park, Insun Yeom, Kyu Won Shim, Mona Choi

**Affiliations:** 1 Mo-Im Kim Nursing Research Institute Yonsei University College of Nursing Seoul Republic of Korea; 2 College of Nursing University of Illinois at Chicago Chicago, IL United States; 3 Department of Pediatric Neurosurgery Yonsei University College of Medicine Seoul Republic of Korea

**Keywords:** affect, ecological momentary assessment, mood, Moyamoya disease, psychological stress

## Abstract

**Background:**

Moyamoya disease (MMD) is a known progressive obstructive cerebrovascular disorder. Monitoring and managing mood and stress are critical for patients with MMD, as they affect clinical outcomes. The ecological momentary assessment (EMA) method is a longitudinal study design by which multiple variable assessments can be performed over time to detect momentary fluctuations and changes in psychological dimensions such as mood and stress over time.

**Objective:**

This study aimed to identify predicting factors associated with momentary mood and stress at both the within-person and between-person levels and to examine individual fluctuation of mood over time in the short term using an EMA method combined with a mobile phone app.

**Methods:**

Participants aged older than 18 years were recruited from a tertiary hospital in Seoul, Korea, between July 2018 and January 2019. The PsyMate scale for negative affect (NA) and positive affect (PA) and the Trier Inventory for Chronic Stress Scale were uploaded on patient mobile phones. Using a mobile app, data were collected four times a day for 7 days. Pearson correlations and mixed modeling were used to predict relationships between repeatedly measured variables at both the between-person and within-person levels.

**Results:**

The mean age of the 93 participants was 40.59 (SD 10.06) years, 66 (71%) were female, and 71 (76%) were married. Participants provided 1929 responses out of a possible 2604 responses (1929/2604, 74.08%). The mean momentary NA and PA values were 2.15 (SD 1.12) and 4.70 (SD 1.31) out of 7, respectively. The momentary stress value was 2.03 (SD 0.98) out of 5. Momentary NA, PA, and stress were correlated (*P*<.001) and varied over time in relation to momentary variables. Common momentary variables associated with momentary mood and stress at both the within-person (level 1) and between-person (level 2) levels were identified. Momentary NA increased when being alone and being at the hospital at both levels, whereas momentary PA increased when eating or drinking, resting, being at a café, restaurant or a public place but decreased when being alone at both levels. Momentary stress increased when being at the office, at a public place, or as the time of the day went by but decreased when resting or during the weekend. Different factors affecting mood and stress at different levels were identified. Fluctuations in individual momentary mood over time at the within-person level were captured.

**Conclusions:**

The EMA method using a mobile phone app demonstrated its ability to capture changes in mood and stress in various environmental contexts in patients with MMD. The results could provide baseline information for developing interventions to manage negative mood and stress of patients with MMD based on the identified predictors affecting mood and stress at two different levels.

## Introduction

### Background

Moyamoya disease (MMD) is a rare idiopathic vascular disorder that is characterized by progressive bilateral stenosis or occlusion of the distal branches of the carotid arteries with an abnormal vascular network [1,2]. It is associated with the development of bifurcation and compensatory arterial collateral networks at the base of the brain [1,2], hence the name *moyamoya* (describing a puff of smoke in Japanese) [1]. MMD is common in Asian countries, such as Korea and Japan, with sparse observation in Europe and the Americas [3,4].

The prevalence of MMD in Korea has gradually increased and reached 16.1 per 100,000 persons in 2011 [4]. This increase can be partly explained by the increasing availability of diagnostic tests, such as magnetic resonance (MR) imaging or MR angiography and prolonged survival owing to improved management [4,5]. In Japan, the prevalence rate was 6.03 per 100,000 persons in 2003 [6], and in Nanjing, China, the prevalence rate was 3.92 per 100,000 persons in 2010 [7]. The incidence rate of MMD in the United States was lower than that in East Asian countries at 0.087 per 100,000 persons [8], whereas moyamoya syndrome (MMS), termed unilateral moyamoya angiopathy [9], is considered to have higher prevalence in Western countries than in East Asian countries [10]. The prevalence of MMS in Western countries was found to be close to that in Japan at 0.34 per 100,000 persons in a nationwide survey [11]. The relatively high prevalence of MMS in Western countries may be related to the fact that sickle cell disease is a frequent cause of MMS in individuals of African origin living in the United States and Europe [12,13].

The clinical features of MMD or MMS in adult patients generally involve cerebral hemorrhages and infarction, whereas children develop ischemic attacks [4]. Relating to the disease’s chronic and uncertain nature, it has been observed that improper management of mood and stress can lead to reduced cerebrovascular blood flow, which is closely related to the prognosis of the disease [14]. According to recent studies, adults with MMD are vulnerable to stress and mood changes [14] and may have anxiety, depression, and posttraumatic stress syndrome [15].

Mood is a state of subjective feeling that can be changed by events and is typically described as having either positive or negative valences [16]. Individual differences exist in experiencing the states of positive and negative feelings, and these are assessed as positive and negative effects [17]. Negative mood states such as depression can extend to psychosocial distress that potentially leads to stress and, more generally, to a negative outlook on life [18,19]. Mood and stress vary with time in relation to the surrounding context, showing changes over time and interindividual differences [20].

The ecological momentary assessment (EMA) method, also known as the experience sample method or ambulatory assessment, is a repeated observational study design by which time-varying variables can be assessed in natural and real-life environments [20-22]. For example, participants self-report their mood or anxiety multiple times to observe changes based on the environment and/or time while performing their ordinary daily tasks, without changing their life patterns to attend a survey [23]. Participants can also report various events, types of food, and calories per serving multiple times per day to track down changes in their eating behavior over days or weeks [24]. Such reports can be completed through diaries, personal digital assistants, mobile apps, or wearable sensors [25] in the short or long term, depending on the study goals and design [26]. This method is reportedly accurate and able to detect changes in psychological properties through multiple daily assessments [27,28], helps address questions regarding individual differences [29], and elucidates momentary changes and fluctuations in psychological dimensions such as mood and stress over time and across situations [30,31]. It has been widely used to assess the psychological characteristics of participants with [32,33] and without [26] mental problems. Recently, various tools using mobile phone technology have been developed and used to measure mood and stress in diverse patient populations [26]; thus, this study utilized the EMA approach for moyamoya patients’ condition.

### Objectives

This study aimed to identify predicting factors associated with momentary mood and stress at both the within-person and between-person levels and to examine individual fluctuation of mood over time in the short term using an EMA method combined with a mobile phone app.

## Methods

### Participants

Adult patients with MMD, who visited the outpatient clinic of a tertiary hospital or were admitted in the same hospital, were recruited from July 2018 to January 2019. Only participants who used Android operating systems were included, as the developed mobile app was only available for this operating system with the version 4.4 or higher as described in a previous study [32]. To exclude possible cognitive impairment that would preclude the participants from answering the questionnaire, all patients were required to have a score higher than 24 on the Korean version of the Mini-Mental State Examination [33], which is widely used to test the cognitive ability of clinical populations in Korea. This study was approved by the institutional review board of the Yonsei University Health System (approval number: 4-2018-0385), and informed consent was obtained from each participant.

### Measurements

Measurements in this study included baseline variables, such as demographic characteristics, disease-specific information, trait mood (anxiety and depression), and trait stress. Momentary measures were mood and stress. The study variables at baseline and momentary measures are summarized in Table 1.

**Table 1:** Study variables at all time points.

Variables	Baseline	Ecological momentary assessment						
		Day 1	Day 2	Day 3	Day 4	Day 5	Day 6	Day 7
Demographic characteristics	x	N/A^a^	N/A	N/A	N/A	N/A	N/A	N/A
Disease-specific information	x	N/A	N/A	N/A	N/A	N/A	N/A	N/A
Korean Hospital Anxiety and Depression Scale	x	N/A	N/A	N/A	N/A	N/A	N/A	N/A
Korean Perceived Stress Scale	x	N/A	N/A	N/A	N/A	N/A	N/A	N/A
Momentary mood (PsyMate)	N/A	x	x	x	x	x	x	x
Momentary stress (Trier Inventory for Chronic Stress)	N/A	x	x	x	x	x	x	x

^a^x: variables measured at the day point.

^b^N/A: not applicable.

### Baseline Measures

Patients provided demographic information such as age, sex, income, level of education, symptoms experienced, and disease duration since the diagnosis. The perceived severity of the disease was also self-reported at baseline using a 5-point scale.

We used the Korean version of the Hospital Anxiety and Depression Scale (K-HADS) [34] to measure trait mood after obtaining the permission of the scale provider. The HADS is known as a reliable and valid scale and used worldwide for measuring mental health in clinical settings [35]. The Cronbach alphas of the K-HADS anxiety and depression subscales have been reported to be .89 and .86, respectively [35]. The scale consists of 14 items, 7 items for assessing anxiety and 7 items for depression, measured on a 4-point scale, from 0 to 3. A higher score denotes a higher level of anxiety or depression. The Cronbach alphas for the anxiety and depression subscales in this study were .75 and .81, respectively.

To measure trait stress, we used the Korean version of the Perceived Stress Scale, which consists of 10 items [36]. This scale was freely downloaded from the official homepage of the Laboratory for the Study of Stress, Immunity, and Disease of the Carnegie Mellon University. It has negative and positive subdomains and five negative items for stress and five positive items for coping ability rated on a 5-point Likert scale (0–4). The Cronbach alphas of the two subdomains have been reported to be .87 and .71, respectively [36]. The Cronbach alpha in this study was .89 for both subdomains.

### Momentary Measures Using a Mobile App

Momentary mood was measured using the Korean version of the PsyMate, translated from the English version with reference to the original Dutch version. We obtained permission to use PsyMate from the developers [37]. It consists of nine items assessing negative affect (NA) and four items assessing positive affect (PA). The Cronbach alphas of the subscales of NA and PA were .91 and .92, respectively, in the previous study [37]. The Cronbach alphas in this study were .94 and .92 for NA and PA, respectively.

Stress was assessed by the Korean version of the Trier Inventory for Chronic Stress, adapted from the German version [38,39]. It consists of eight items measuring work overload, social overload, pressure to perform, work discontent, excessive demands from work, lack of social recognition, social tensions, and social isolation. The Cronbach alpha in this study was .79.

The questionnaires were uploaded on a mobile app for the Android operating system developed in the previous study [32]. Momentary mood and stress were measured in an environmental context considering what the participants were doing, where they were, and with whom they were at the moment of answering.

### Procedure

After obtaining participant informed consent, we held an individual and face-to-face 30-min intake session with each patient. Patients filled the baseline measures and were allowed time to download the app and practice answering for the EMA study. Researchers helped the participants answer the baseline survey and install the app. Patients were provided a reward in coupons when they completed the baseline survey and enrollment. They were also informed that they would receive additional coupons on completion of the EMA study. Researchers preset the survey period for each patient in advance to push notifications and instructed participants to carry their mobile phone during the scheduled survey period and to answer the survey question when they received the notification requesting them to do so.

Measures of mood and stress were set on the mobile app, and notifications were set to appear four times a day for 7 consecutive days (4 times ×7 days=28 times/person) in semirandom, 90-min blocks. Notifications were sent in the morning between 8 AM and 9 PM, early afternoon between noon and 1 PM, evening between 5 PM and 6 PM, and at night between 9 PM and 10 PM. Participants were instructed that they would receive a reminder notification when they did not input the response within 45 min after they received the first notification for each scheduled measurement. Researchers monitored participant compliance to the protocol and managed participation by phoning patients who did not respond on the first day and attempted to solve any participation difficulties and problems in the EMA study.

### Statistical Analysis

Data analysis was performed with STATA 14.0 (StataCorp) using 1929 responses from 93 participants who provided more than three responses in the total course of the study to capture changes over time [20]. We analyzed participant characteristics by descriptive analysis. Independent *t* tests and analysis of variance were used to compare the mean differences between the variable groups. We estimated the Pearson coefficient to examine the correlation between the momentary variables. Mixed modeling analysis, handling the clustered and correlated data [20], was used to describe and predict factors associated with momentary NA, PA, and stress at both the within-person (level 1) and between-person (level 2) levels. The threshold of statistical significance was set at *P*<.05.

## Results

### Participants

A total of 93 participants with MMD were recruited. Of 93 participants, 71 (76%) were recruited from the outpatient department and 22 participants (24%) were recruited from the admission wards of a university hospital. The mean age of the participants was 40.59 years (SD 10.06), 71% (66/93) participants were female, and 76% (71/93) participants were married. The mean number of years since diagnosis was 3.68 (SD 4.05), and the perceived severity level was 3.56 (SD 1.00) out of 5. The mean HADS anxiety and depression scores were 7.17 (SD 3.38) and 7.14 (SD 3.51) out of 21, respectively, and the mean perceived stress level was 1.64 (SD .98) out of 4. The participant baseline characteristics are summarized in Table 2.

**Table 2:** Participant characteristics at baseline (n=93).

Characteristics			n (%)		Mean (SD)		Possible range
**Age (years)**							
	20–29	11 (12)		40.59 (10.06)		N/A^a^	
	30–39	37 (40)		N/A		N/A	
	40–49	26 (28)		N/A		N/A	
	50–59	15 (16)		N/A		N/A	
	≥60	4 (4)		N/A		N/A	
**Sex**							
	Female	66 (71)		N/A		N/A	
	Male	27 (29)		N/A		N/A	
**Marital status**							
	Married	71 (76)		N/A		N/A	
	Not married	22 (24)		N/A		N/A	
**Education**							
	≤High school	41 (44)		N/A		N/A	
	≥College	52 (56)		N/A		N/A	
**Monthly household income^b^ (US $)**							
	<2000	23 (25)		N/A		N/A	
	2000–3000	16 (18)		N/A		N/A	
	3000–4000	22 (24)		N/A		N/A	
	>4000	31 (33)		N/A		N/A	
Years since the diagnosis			N/A		3.68 (4.05)		N/A
Perceived severity			N/A		3.56 (1.00)		1–5
HADS^c^ anxiety			N/A		7.17 (3.38)		0–21
HADS depression			N/A		7.14 (3.51)		0–21
Perceived stress			N/A		1.64 (.98)		0–4

^a^N/A: not applicable.

^b^n=92.

^c^HADS: Hospital Anxiety and Depression Scale.

### Momentary Responses

Participants provided 1929 responses out of a possible 2604 (1929/2604, 74.1%). Of 1883 responses, 799 (42.4%) were answered while participants were resting, 344 responses (18.3%) while working, 293 responses (15.6%) doing household work, and 188 responses (10.0%) while eating or drinking at the moment of answering. Of the 1929 responses, 1147 (59.5%) were obtained when participants were at home, 325 responses (16.9%) were obtained at the office, and 87 responses (4.5%) were obtained at a café or restaurant. Of all 1929 responses, 444 (23.1%) were obtained while the participants were alone, and 1348 (69.9%) were obtained on weekdays. Of 1929 responses, 489 (25.3%), 497 (25.8%), 509 (26.4%), and 434 (22.5%) were provided in the morning, afternoon, evening, and at night, respectively. The mean momentary NA and PA were 2.15 (SD 1.12) and 4.70 (SD 1.31) out of 7, respectively. The mean momentary stress level was 2.03 (SD .68) out of 5. Measures of momentary variables are summarized in Table 3.

**Table 3:** Measures of momentary variables (n=1929).

Momentary variables			n (%)		Mean (SD)		Possible range
**What^a^ (things doing)**							
	Household work	293 (15.6)		N/A^b^		N/A	
	Working	344 (18.3)		N/A		N/A	
	Eating/drinking	188 (10.0)		N/A		N/A	
	Resting	799 (42.4)		N/A		N/A	
	Other	259 (13.7)		N/A		N/A	
**Where^c^ (place being)**							
	Home	1147 (59.5)		N/A		N/A	
	Office	325 (16.9)		N/A		N/A	
	Café or restaurant	87 (4.5)		N/A		N/A	
	Hospital	66 (3.4)		N/A		N/A	
	Public place	138 (7.2)		N/A		N/A	
	Other	164 (8.5)		N/A		N/A	
**Being alone**							
	Yes	444 (23.1)		N/A		N/A	
	No	1485 (76.9)		N/A		N/A	
**Weekend**							
	Yes	581 (30.1)		N/A		N/A	
	No	1348 (69.9)		N/A		N/A	
**Time of day**							
	Morning (8 AM to 9 PM)	489 (25.3)		N/A		N/A	
	Afternoon (12 noon to 1 PM)	497 (25.8)		N/A		N/A	
	Evening (5 PM to 6 PM)	509 (26.4)		N/A		N/A	
	Night (9 PM to 10 PM)	434 (22.5)		N/A		N/A	
Momentary negative affect			N/A		2.15 (1.12)		1–7
Momentary positive affect			N/A		4.70 (1.31)		1–7
Momentary stress			N/A		2.03 (0.68)		1–5

^a^n=1883.

^b^N/A: not applicable.

^c^n=1927.

**Correlations Between Momentary Negative Affect, Positive Affect, and Stress**

Momentary NA, PA, and stress were significantly correlated (*P*<.001). Momentary NA was negatively correlated with momentary PA (*r*=−0.607; *P*<.001) and positively with momentary stress (*r*=0.538; *P*<.001). Momentary PA was negatively correlated with momentary stress (*r*=−0.272; *P*<.001). The correlation coefficients between variables are presented in Table 4.

**Table 4:** Correlation coefficients between momentary variables (n=93).

Momentary variables	Momentary NA^a^ (r value)	Momentary PA^b^ (r value)
Momentary NA	1	-0.607^c^
Momentary PA	−0.607^c^	1
Momentary stress	0.538^c^	−0.272^c^

^a^NA: negative affect.

^b^PA: positive affect.

^c^*P*<.001.

**Modeling of Between-Person and Within-Person Analysis**

Analysis by mixed modeling was performed based on 1929 completed assessments from the participants who provided more than three responses for analyzing changes of momentary mood and stress over time at both level 1 (within-person) and level 2 (between-person).

Momentary predicting factors were examined by three models at levels 1 and 2. Disease-specific variables such as perceived severity and years since the diagnosis, age, sex, and trait mood variables at the baseline were added into models 2 and 3 for adjustment. Momentary variables included *things doing* (what), *place being* (where), *being alone* or not (with whom), answering during the *weekend* or not, and *time of day*. Table 5 shows the variables included in each model at both the within-person and between-person levels.

**Table 5:** Designed levels, models, and variables.

Level and model		Variables
**Within-person**		
	1. Momentary variables	What, where, with whom, weekend, and time of day
	2. Disease-specific variables	Variables of Model 1 + perceived severity and years since the diagnosis
	3. Trait variables	Variables of Model 2 + age, sex, trait anxiety/depression, and stress
**Between-person**		
	1. Momentary variables	What, where, with whom, weekend, and time of day
	2. Disease-specific variables	Variables of Model 1 + perceived severity and years since the diagnosis
	3. Trait variables	Variables of Model 2 + age, sex, trait anxiety/depression, and stress

**Momentary Variables Affecting Momentary Mood and Stress at the Within-Person and Between-Person Levels**

Common momentary variables associated with momentary mood and stress at both the within-person (level 1) and between-person (level 2) levels were identified. Momentary NA increased when being alone and being at the hospital at both levels, whereas momentary PA increased when eating or drinking, resting, being at a café or restaurant, or at the public place but decreased when being alone at both levels. Momentary stress increased when at the office, at the public place, or as the time of the day went by but decreased when resting or during the weekend.

Different factors affecting momentary mood and stress at different levels were also identified. Variables of being at a café or restaurant (coefficient=−0.19; *P*=.03) and during the weekend (coefficient=−.08; *P*=.03) were associated with momentary NA at the within-person level only. However, eating or drinking (coefficient=−0.20; *P*=.04) and resting (coefficient=−0.21; *P*=.01) were associated with momentary NA at the between-person level only. There was no difference in factors associated with momentary PA either at the within-person or at the between-person level. Among variables, being at the hospital (coefficient=5.67; *P*<.001) was associated with momentary stress at the between-person level but not at the within-person level. Table 6 shows the parameter estimates from the mixed effect model at both levels.

**Table 6:** Fixed effect model parameter estimates at within-person and between-person levels in model 3.

Variables^a^		Momentary negative affect, coefficient (SE)		Momentary positive affect, coefficient (SE)			Momentary stress, coefficient (SE)			
		Within	Between	Within	Between		Within	Between		
**What (things doing)**										
	Household work	Reference	Reference	Reference	Reference	Reference		Reference		
	Working	0.05 (0.09)	0.08 (0.12)	−0.20 (0.12)	−0.20 (0.15)	0.26 (0.42)		0.64 (0.61)		
	Eating or drinking	−0.09 (0.07)	−0.20 (0.10)^b^	0.24 (0.09)^b^	0.27 (0.12)^b^	0.23 (0.33)		−0.56 (0.49)		
	Resting	−0.08 (0.05)	−0.21 (0.07)^c^	0.15 (0.06)^b^	0.30 (0.08)^c^	−1.03 (0.23)^c^		−1.56 (0.33)^c^		
	Other	−0.01 (0.06)	−0.02 (0.09)	0.03 (0.09)	−0.05 (0.11)	−0.26 (0.31)		−1.20 (0.45)		
**Where (place being)**										
	Home	Reference	Reference	Reference	Reference	Reference		Reference		
	Office	0.05 (0.08)	−0.19 (0.12)	−0.04 (0.11)	0.21 (0.14)	1.82 (0.41)^c^		1.56 (0.58)^c^		
	Café or restaurant	−0.19 (0.09)^b^	−0.10 (0.12)	0.37 (0.12)^c^	0.33 (0.15)^b^	−0.75 (0.42)		−0.17 (0.61)		
	Hospital	0.32 (0.12)^b^	0.54 (0.12)^c^	−0.17 (0.16)	−0.07 (0.15)	0.60 (0.58)		5.67 (0.62)^c^		
	Public place	−0.06 (0.07)	0.01 (0.09)	0.21 (0.09)^b^	0.28 (0.12)^b^	0.91 (0.33)^c^		2.13 (0.47)^c^		
	Other	−0.10 (0.06)	−0.10 (0.09)	0.22 (0.08)	0.35 (0.11)	0.08 (0.30)		0.83 (0.43)		
Being alone		0.10 (0.04)^b^	0.24 (0.05)^c^	−0.16 (0.05)^c^	−0.49 (0.07)^c^	−0.37 (0.20)		−0.34 (0.27)		
Weekend		−0.08 (0.03)^b^	−0.03 (0.05)	0.02 (0.05)	0.01 (0.06)	−1.20 (0.16)^c^		−0.76 (0.25)^c^		
**Time of day**										
	8-9 AM	Reference	Reference	Reference	Reference	Reference		Reference		
	12 noon-1 PM	−0.03 (0.04)	−0.01 (0.06)	0.09 (0.06)	0.08 (0.08)	0.76 (0.20)^c^		0.77 (0.31)^c^		
	5-6 PM	−0.04 (0.04)	−0.01 (0.06)	0.03 (0.05)	−0.03 (0.08)	1.07 (0.20)^c^		1.03 (0.30)^c^		
	9-10 PM	−0.04 (0.04)	−0.05 (0.06)	0.17 (0.06)^c^	0.14 (0.08)	1.48 (0.21)^c^		1.40 (0.32)^c^		
Perceived severity		0.11 (0.08)	0.13 (0.02)^c^	−0.03 (0.09)	−0.06 (0.03)^b^	0.69 (0.41)		0.73 (0.11)^c^		
Years since the diagnosis		−0.03 (0.02)	−0.01 (0.01)	−0.03 (0.02)	−0.04 (0.01)^b^	−0.16 (0.10)		−0.10 (0.03)^c^		
Trait anxiety and depression		0.06 (0.02)^c^	0.07 (0.01)^c^	−0.06 (0.02)^c^	−0.08 (0.01)^c^	0.21 (0.08)^b^		0.19 (0.02)^c^		
Trait stress		0.04 (0.02)^b^	0.03 (0.01)^c^	0.01 (0.02)	0.02 (0.01)^b^	0.22 (0.12)		0.23 (0.03)^c^		

^a^Age and sex adjusted.

^b^*P*<.05.

^c^*P*<.01.

### Individual Fluctuation of Negative Affect and Positive Affect Over Time

**Figure 1:**
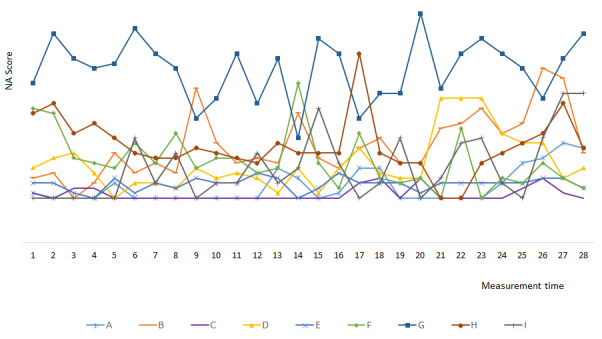
Individual fluctuation of negative affect over time for the selected participants. NA: negative affect; A to I: selected participants.

**Figure 2:**
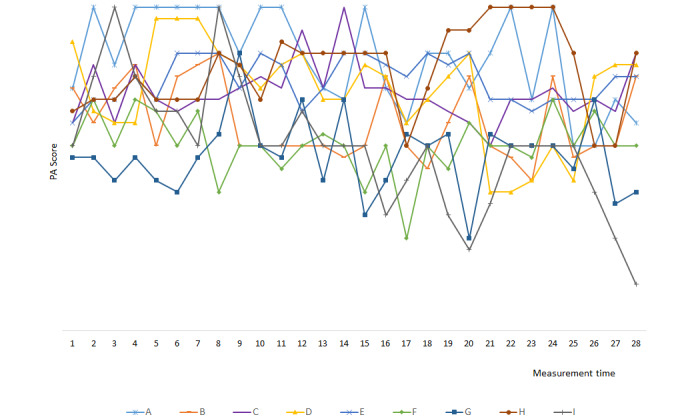
Individual fluctuation of positive affect over time for the selected participants. PA: positive affect; A to I: selected participants.

Both the momentary NA and PA of participants fluctuated over time. We arbitrarily selected 10 participants from those who completed all 28 assessments to show the individual fluctuation of affect over time. Specific graphs of NA and PA fluctuation were constructed for each selected participant in accordance with momentary and trait variables (Figures 1 and 2 as examples).

## Discussion

### Principal Findings

This study used the EMA method to assess and predict momentary factors associated with momentary mood and stress in a real-life context in adult patients with MMD. The results showed that context variables of the participants’ natural environment affected momentary mood and stress at both the within-person and between-person levels after adjusting for participant demographics and disease-specific characteristics and trait anxiety and depression.

We also identified common variables affecting momentary mood and stress at both the within-person and between-person levels and distinguished different variables having different effects at the within-person and between-person levels. Participants commonly showed higher NA when being alone or at the hospital at both levels. Meanwhile, some factors such as activities of eating or drinking or resting affected momentary NA at the between-person level only. Participants commonly expressed decreased stress when resting or during the weekend and increased stress at the office and as the time of the day went by at both levels. Meanwhile, being at the hospital was associated with higher stress only at the between-person level. These results showed that different factors at different levels are associated with NA and stress, although there are numerous common factors. These factor differences between the two levels should be considered when designing individualized interventions to manage the mood and stress of patients with MMD.

This study also established that an EMA method using a mobile app could capture individual fluctuations of mood and stress over time while participants perform their usual daily tasks. This result is aligned with the result from a previous study that applied an EMA app using the same scale of the PsyMate to assess the mood of Dutch patients in an ambulatory mental health setting [38], presenting the ability to detect changes in mood over time.

In an additional analysis, we found that patients who had been diagnosed less than a year ago appeared to be more depressed than those who were diagnosed more than a year ago. These results indicate that an emotional care plan with close, regular monitoring is needed for patients with MMD, especially for those with higher anxiety and depression, and within a year after diagnosis. The levels of anxiety and depression of the participants in this study at baseline were 7.17 (SD 3.38) and 7.14 (SD 3.51) out of 21, respectively. This implies that adults with MMD may also strive to overcome negative feelings, as do patients with other cerebrovascular diseases who are at continuous risk of cerebrovascular hemorrhage and infarction [40,41].

Perceived stress is a known predictor of depression and depressive symptoms in patients with stroke [42,43]. The results of this study also showed that both trait and momentary stress are significantly associated with momentary mood at both the within-person and between-person levels. Stress should be managed, as it triggers negative mood. Negative mood and stress in patients with cerebrovascular disorders are related to emotional distress, which threatens health behaviors and drives patients to avoid health-promoting activities [44].

Perceived social support plays a critical role in buffering stress and promoting psychological well-being [45-47]. Patients with MMD should be encouraged to engage in social interactions with family or self-support groups, as it was shown that being alone is significantly related to momentary increase in negative mood at both levels.

### Limitations and Future Directions

Our study had limitations. This study included patients who used Android OS, and those who used other systems were excluded. In addition, there might be challenges regarding technical issues and potential malfunctioning of the configuration, although a helpline was provided by our research team. These technical points need to be addressed to improve the EMA survey in the future. In addition, as the patients were recruited from a tertiary-level hospital in Seoul, patients in communities or in smaller facilities may differ from this study population in terms of clinical severity, years since the diagnosis, or trait mood and stress levels.

Studies on the impact of social support or stress-coping strategies on mood and stress in MMD warrants further investigation. EMA methods integrated into momentary interventions for improving mood and stress could be a promising future direction in MMD.

### Conclusions

In this study, we evaluated the EMA method using a mobile phone app and demonstrated that the EMA method was able to capture mood and stress change over time and by assessing momentary contextual variables. With the identified predictors affecting mood and stress at two different levels, the results of this study could provide valuable information for developing individualized patient-centered interventions for managing the mood and stress of patients with MMD who are psychologically vulnerable and whose states cannot be easily assessed in a real-world environment.

## Abbreviations

EMA: ecological momentary assessment
K-HADS: Korean version of the Hospital Anxiety and Depression Scale
MMD: Moyamoya disease
MMS: Moyamoya syndrome
MR: magnetic resonance
NA: negative affect
PA: positive affect
